# Collagenated Xenogeneic Versus Autogenous Bone Grafts for Ridge Reconstruction: A Prospective Study

**DOI:** 10.1155/ijod/4758496

**Published:** 2026-08-27

**Authors:** Reem Al Matrooshi, Zaid Hamdoon, Ahmed Aziz, Shishir Shetty, Waseem Jerjes, Waad Kheder

**Affiliations:** ^1^ Emirates Health Service, Sharjah, UAE; ^2^ Department of Oral and Craniofacial Health Sciences, College of Dental Medicine, University of Sharjah, Sharjah, UAE, sharjah.ac.ae; ^3^ Al Bayan University, College of Dental Medicine, Baghdad, Iraq; ^4^ Department of Restorative Dentistry, College of Dental Medicine, University of Sharjah, Sharjah, UAE, sharjah.ac.ae; ^5^ Faculty of Medicine, Imperial College London, London, UK, imperial.ac.uk

**Keywords:** alveolar ridge, customized, ridge reconstruction, xenogeneic bone

## Abstract

This study aimed to compare the clinical performance of autogenous bone blocks with customized and noncustomized bovine bone blocks for horizontal alveolar ridge augmentation. Forty‐eight participants with deficient alveolar ridges were divided into threegroups (*n* = 16): Group I (autogenous bone graft), Group II (noncustomized bovine bone block), and Group III (customized bovine bone block). Graft success, postsurgical complications, bone formation, and resorption rates were recorded and analyzed after 6 months. Patient satisfaction and postoperative pain were assessed using a visual analog scale. A total of 39 bone blocks were integrated successfully. Survival rates for Groups I, II, and III were 100% (95% CI: 79.4%–100%), 75.0% (95% CI: 47.6%–92.7%), and 68.8% (95% CI: 41.3%–89.0%), respectively (*p* = 0.07). Dehiscence, screw exposure, and partial bone loss were the most common postoperative complications. Bone formation and resorption did not differ significantly between groups (*p* = 0.61 and *p* = 0.12, respectively). Group I experienced significantly higher pain from day 3 to day 7 compared to other groups (*p*  < 0.001), with peak pain on day 6 (5.75 ± 1.12), while Groups II and III reported peak pain on day 3 (3.06 ± 0.92 and 3.44 ± 0.81, respectively). By day 10, pain scores had decreased substantially in all groups, with Group III reporting the lowest scores (0.31 ± 0.48, *p* = 0.003). Patient satisfaction was highest in Group III (91%). Within the limitations of this prospective comparative study, onlay grafting with bovine bone blocks demonstrated clinically acceptable outcomes for horizontal ridge augmentation, with resorption rates comparable to autogenous grafts. However, the observed survival differences did not reach statistical significance, and the wide confidence intervals reflect the limited sample size, suggesting that clinically meaningful differences cannot be excluded. Customized bovine bone blocks showed similar clinical performance to noncustomized blocks with higher patient satisfaction. Larger randomized controlled trials with longer follow‐up are warranted to confirm these findings.

## 1. Introduction

Teeth loss results in progressive and irreversible horizontal and vertical bone resorption. The bone begins to resorb immediately after tooth loss as the physiological stimulus that keeps the bone under the continuous remodeling cycle disappears [1]. Local factors such as traumatic dental extractions, systemic factors such as nutritional abnormalities, and other hormonal and genetic factors may affect bone metabolism, leading to accelerated bone resorption and eventually fracture of the jaw [1, 2]. A strong correlation between the time of extraction and degree of bone resorption has been established; vertical and horizontal ridge resorption reached 11%–22% and 29%–63%, respectively, within 6 months [3]. Moreover, the anatomic features of the bundle bone in the buccal/labial wall resulted in higher resorption than on the lingual or palatal side [4, 5].

Bone resorption varies in degree and shape, but horizontal bone deficiency represents the most clinically relevant form [6]. This challenge has led to the use of diverse techniques and the choice of multiple materials to augment deficient bones. Various materials from different origins have been proposed, including autogenous graft material, allografts [7, 8], xenografts [9], and synthetic and composite bone grafts [10–12]. Several surgical techniques have been described for reconstructing alveolar ridge dimensions, including guided bone regeneration (GBR), ridge splitting, and bone block grafting [13]. Autogenous graft material represents the criterion standard in alveolar bone regeneration due to the presence of osteogenic cells, biocompatibility, Oste inductivity, and osteoconductivity [14, 15]. However, donor site morbidity, graft adaptation, and a high reporting rate render this technique challenging [16].

Particulate grafts are advantageous in terms of adaptability to irregular defects and enhanced surface area for revascularization; however, they are more dependent on containment membranes and are susceptible to particle displacement and soft tissue collapse in large defects. In contrast, block grafts provide structural stability, superior space maintenance, and resistance to soft tissue pressure, which is particularly important in cases of severe horizontal ridge deficiency. In addition, block grafts contribute to improved blood stabilization and maintain a three‐dimensional scaffold that may facilitate more predictable bone regeneration in compromised alveolar sites. For these reasons, block grafts were selected in the present study to ensure adequate dimensional stability and reproducible outcomes in horizontal ridge augmentation.

With the advent of cone beam computed tomography (CBCT) and computer‐aided design and computer‐aided manufacturing (CAD/CAM) technology, customized nonautogenous bone grafting has become a treatment option. Several case reports and research have demonstrated high‐precision fit and the successful application of customized nonautogenous blocks [17, 18]. However, a few reports have compared different graft designs with the criterion standard autogenous bone block [19–21]. In a recent consensus statement, they concluded that allogenic bone replacement grafts provided similar mechanical properties to the autologous bone and might contain the collagenous matrix and proteins of natural bone, even though they lacked viable cells. In addition, they outlined that the handling properties were comparable with autologous bones. However, possible disease transmission, potential unwanted immune reactions, variation in resorption rates, and possible impairment of the grafted site have been reported. In contrast, bovine bone graft has less antigenic potential and better clinical behavior. Despite their widespread use, xenografts are associated with certain limitations. These include slower and variable resorption rates, limited osteoinductive and osteogenic potential, and dependence on osteoconductive properties alone for bone regeneration. In addition, concerns have been raised regarding potential immunogenic reactions and, although rare, theoretical risks of disease transmission [22].

The authors are unaware of a previous clinical study comparing the clinical outcomes of customized and noncustomized bovine bone blocks with autogenous bone graft in different anatomical locations. Therefore, the aim of this clinical study was to compare the clinical performance of autograft bone blocks with customized and noncustomized bovine bone blocks for horizontal alveolar ridge augmentation in four anatomical areas.

## 2. Material and Methods

This prospective clinical study was conducted at the University Dental Hospital of Sharjah between September 2021 and June 2023. The study was conducted in accordance with the ethical standards outlined in the 1964 Declaration of Helsinki and according to the STROBE guidelines. The study was independently reviewed and approved by the research ethics committee (REC‐21‐01‐23‐01‐S).

### 2.1. Study Design

**Figure 1:**
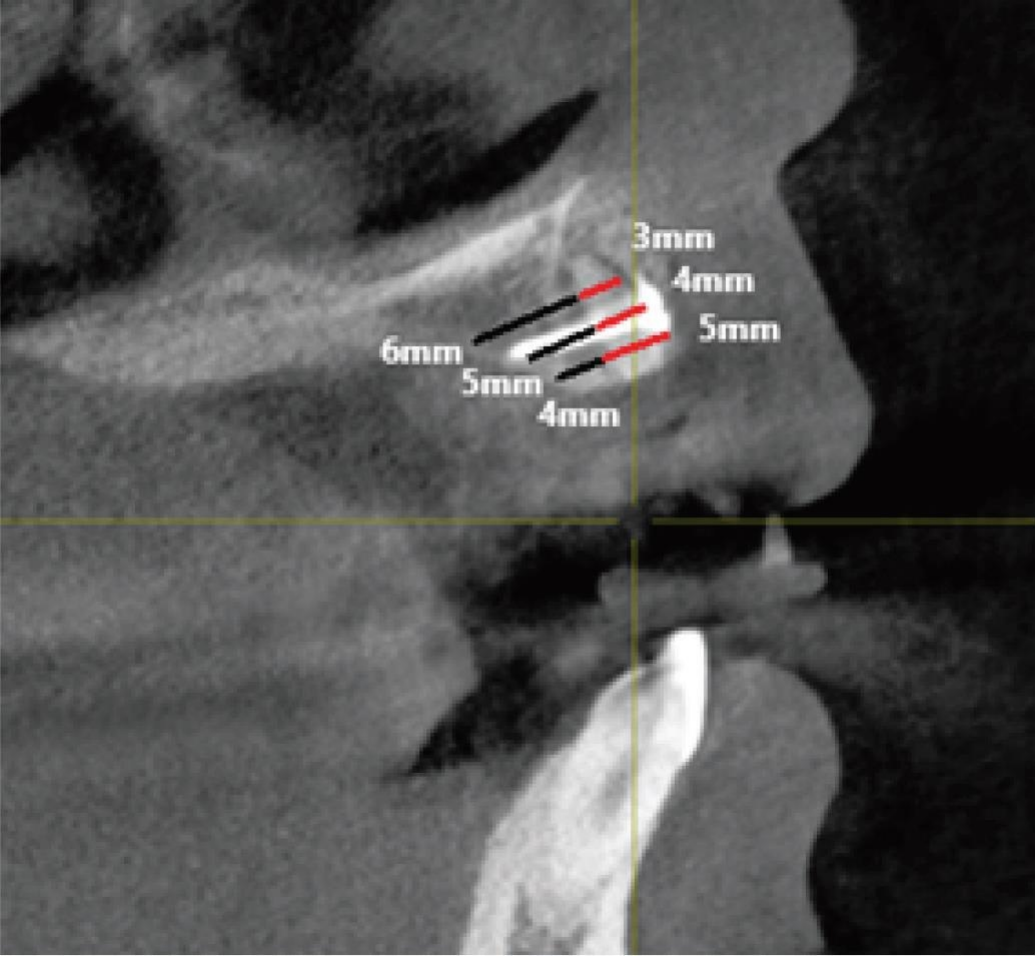
Selection criteria for standardization and uniformity of the included cases. Native bone thickness of average 4to 5 mm thickness at 2, 5, and 8 mm subcrestal areas (black lines) with alveolar bone height of 10 mm is a prerequisite for inclusion. Augmentation of the native bone with bone graft to obtain the final thickness of 9 mm (red line arrow) using the osteosynthesis screw of 10 mm as indicator of measurement.

The study enrolled 48 participants, and demographic information of included participants is listed in Table 1. The participants were evenly distributed across three groups, each consisting of 16 individuals. Group I received autogenous bone blocks, Group II received noncustomized adjusted bovine bone blocks, and Group III received customized bovine bone blocks. Alveolar ridge defects were classified according to the Seibert classification (Seibert, 1983), which describes the morphological pattern of ridge deficiency. All included sites were Class I defects (horizontal deficiency). In addition, residual ridge width was assessed using CBCT to quantify the degree of horizontal bone loss. Defects with a residual ridge width of 4–5 mm were considered moderate horizontal deficiencies. Block grafts were selected in the present study to provide superior three‐dimensional stability, improved space maintenance, and resistance to soft tissue collapse [23]. This approach was chosen to ensure predictable augmentation in cases where long‐term volumetric stability was a primary treatment objective. This ensured that the final ridge thickness would reach 9 mm in all selected cases, maintaining consistency among the groups and reducing confounding factors (Figure 1).

**Table 1:** Demographic information of included participants.

Demographic/clinical variable	*n* (%)
Gender	
Male	31 (64.5)
Female	17 (35.5)
Location	
Anterior maxilla	16 (33.4)
Posterior Maxilla	8 (16.6)
Anterior mandible	6 (12.5)
Posterior mandible	18 (37.5)
ASA	
Type I	33 (68.7)
Type II	15 (31.3)
Reason of extraction	
Periodontal disease	12 (25.0)
Caries	27 (56.3)
Trauma	9 (18.7)
Extraction time	
<1 year	3 (6.3)
2–3 years	8 (16.7)
3–5 years	11 (22.9)
5–10 years	14 (29.1)
>10 years	12 (25)
Gingival biotype	
Scalloped	8 (16.7)
Thin	15 (31.3)
Thick	25 (52.0)

The study included systemically healthy individuals who underwent a preoperative medical assessment based on the American Society of Anaesthesiologists (ASA) Physical Status Classification System. Patients classified as ASA I or ASA II with well‐controlled medical conditions were considered eligible for participation. Inclusion criteria comprised the presence of a horizontal alveolar ridge deficiency requiring the replacement of one or two teeth, a minimum residual vertical ridge height of 10 mm, healthy oral mucosa, and at least 3 mm of keratinized attached gingiva.

Patients were excluded if they had systemic or local conditions that could adversely affect surgical outcomes or impair wound healing. Additional exclusion criteria included smoking or alcohol abuse, insufficient vertical bone height (<10 mm), the need for horizontal ridge augmentation involving more than two adjacent teeth, a history of malignancy treated with chemotherapy or radiotherapy within the preceding 5 years, current or previous use of immunosuppressive medications, bisphosphonates, or high‐dose corticosteroids, and the presence of inflammatory or autoimmune diseases affecting the oral cavity.

### 2.2. Surgical Procedure

**Figure 2:**
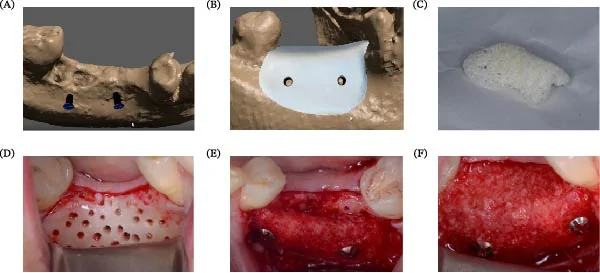
Customized bovine bone block. (A) Preoperative scan of the ridge. (B) Three‐dimensional defect planning and graft designing. (C) Allograft milling and adjusting to the final design (D) Decortication of the outer cortical plate to allow blood supply to the graft. (E) Customized bovine block fixation by two 1.2 × 10 mm osteosynthesis screws. (F) Final clinical outcome 6 months after the surgery with substantial bone formation.

In all cases, a full thick mucoperiosteal flap with two vertical releasing incisions was elevated to provide adequate access to the recipient site. Following flap reflection, cortical decortication was performed using a small round bur or a 0.9 mm drill to create multiple perforations in the recipient bone. This was performed in all groups to enhance local bleeding, improve revascularization, and promote graft integration (Figure 2).

#### 2.2.1. Group I (Autogenous Bone Blocks)

The donor bone was harvested as a cortico‐cancellous block from the chin in all cases. The dimensions of the harvested bone block were determined according to the length of the defect, ensuring adequate coverage of the recipient site with a minimum thickness of 5 mm after adaptation. For single‐tooth defects, a graft size of 10 × 8 mm was used, whereas defects involving two adjacent teeth required a 20 × 10 mm graft. The grafts were shaped to a final thickness of 4–5 mm and adapted to the recipient site to maximize bone‐to‐bone contact. Fixation was achieved using titanium osteosynthesis screws (1.2 mm diameter and 10 mm length), with a minimum of two fixation points per graft. If primary stability was not achieved, a 1.4 mm screw (Bionnovation bone screw) was used. The graft margins were carefully contoured to ensure a smooth transition with the surrounding bone and covered with a platelet‐rich fibrin (PRF) membrane.

#### 2.2.2. Group II (Noncustomized Bovine Bone Blocks)

The bovine bone block used in this study was a mineralized, deproteinized inorganic bone matrix composed of hydroxyapatite and collagen types I and III, preserving a natural cancellous architecture. This biomaterial is derived from bovine sources and is classified as a Class III medical device under Directive 93/42/EEC (Rule 8: implantable devices). Following flap elevation and decortication, the recipient site was exposed and prepared. A single collagenated xenogeneic bone block (CXBB) measuring 5 × 10 × 10 mm was used for single‐tooth defects, whereas larger defects required a 5 × 20 × 20 mm block (Bionnovation Bonefill). The blocks were shaped intraoperatively to match the defect morphology and fixed with titanium osteosynthesis screws to ensure stability. A bilayer PRF membrane was placed over the grafted area to enhance soft tissue healing and protection.

#### 2.2.3. Group III (Customized Bovine Bone Blocks)

**Figure 3:**
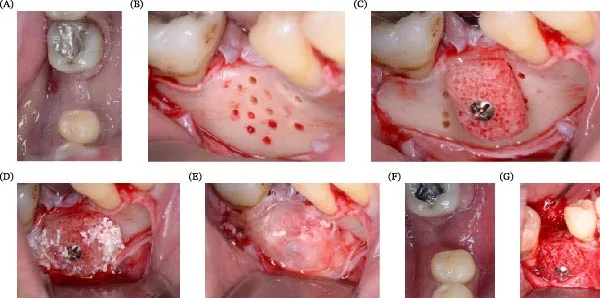
Horizontal bone augmentation with bovine bone block (A). Preoperative ridge before augmentation (B). Three‐sided flap for bone exposure and decortication of the outer cortical plate (C). Bovine block fixation by 1.2 × 10 mm osteosynthesis screw (D). Particulate bovine chips filling the gaps between the bovine block and the native bone (E). Graft coverage by Platelet Rich Fibrin PRF membrane (F). Final clinical outcome 6 months after the surgery with substantial bone formation (G).

The customized bone blocks were fabricated from the same bovine source as Group II. Preoperative CBCT scans were exported in DICOM format and transferred to a digital laboratory workflow. The files were processed and converted into STL format for virtual design and manufacturing. Graft dimensions, including thickness and extension, were customized based on the residual alveolar bone morphology. The blocks were designed with pre‐planned screw channels to facilitate passive adaptation and reduce the risk of fracture during fixation. Following flap elevation and decortication, the customized blocks were positioned and secured using titanium screws. A bilayer PRF membrane was applied over the grafted site (Figure 3), and primary closure was achieved using 4‐0 resorbable polyglycolic acid sutures (Resoquick‐Resorba).

### 2.3. Postoperative Protocol

Postoperative instructions included gentle tooth brushing and rinsing with 0.12% chlorhexidine twice daily for 14 days. Patients received antibiotics for 7 days (amoxicillin 500 mg or clindamycin 300 mg every 8 h) and ibuprofen 600 mg four times daily for pain control. Sutures were removed after 2 weeks. Follow‐up visits were scheduled at 1 week, 2 weeks, 1 month, 3 months, and 6 months to assess complications, including pain, infection, discomfort, graft mobility, graft exposure, and screw exposure. Pain levels were recorded using a visual analog scale (VAS).

### 2.4. Clinical and Radiographical Evaluation

The participants underwent CBCT scanning using the Galileos 3D X‐ray system (Sirona Dental Systems), with imaging restricted to the region of interest to minimize radiation exposure. The grafted sites were evaluated three times: preoperatively (baseline), immediately postoperatively, and at 6 months follow‐up. Ridge width measurements were primarily obtained from CBCT images at each time point. In addition, during surgery, ridge thickness was verified intraoperatively using a sterile bone caliper after flap reflection to confirm agreement with preoperative CBCT measurements and ensure accurate graft adaptation. At each imaging interval, the alveolar bone width was independently assessed by a calibrated radiologist and an oral surgeon to enhance measurement reliability and reduce interobserver variability. A training session was conducted for the readers to achieve a high level of interobserver agreement (κ = 0.95).

**Figure 4:**
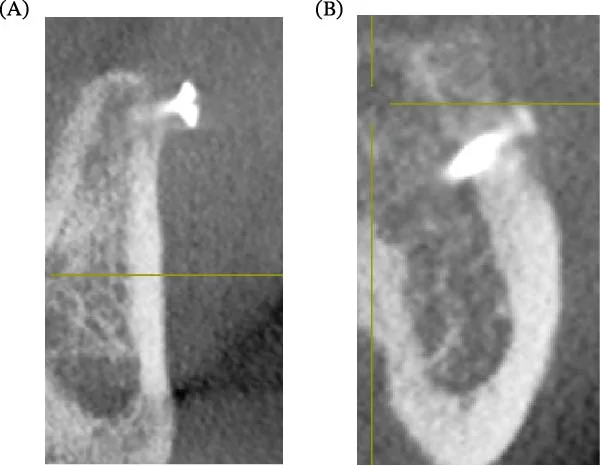
Sectional CBCT scan in coronal section showing the head of the osteosynthesis screw used as reference. (A) Significant bone resorption represented by bone shadow between the internal side of screw head and the superior surface of the bone graft. (B) Stable bone with zero bone resorption around the head of the screw.

Horizontal linear measurements were conducted by referencing three specific points along the defect: 2 mm below the crest (crestal), 5 mm below the crest (mid‐crestal), and 8 mm below the crest (apical). These measurements were used to assess the changes in bone thickness over time. The average bone thickness from baseline and 6‐month readings was calculated to compare the outcomes and assess bone regeneration. The head and shaft of the fixation screws served as consistent reference points to ensure the reproducibility of radiographic measurements and reduce subjectivity throughout the study. The screw head remaining in contact with the superior surface of the bone graft was indicative of zero bone resorption (Figure 4).

### 2.5. Outcome Measures

The primary outcomes of the study were graft success at 6 months, defined as stable graft integration with surrounding native bone, absence of radiolucent lines at the graft–host interface, and no clinical signs of failure (i.e., wound dehiscence, graft exposure, infection, mobility, or necrosis). Secondary outcomes included radiographic and clinical parameters of bone regeneration (bone formation and resorption), postoperative complications, sensory changes in the grafted area, and patient‐reported outcomes. Postoperative follow‐up was conducted for up to 6 months to assess the primary outcomes. Graft failure was defined as progressive wound dehiscence, graft mobility, purulent discharge, or total exposure to necrosis and contamination.

Clinical complications were recorded, including wound dehiscence, graft exposure, partial graft loss, screw exposure, and graft mobility. Sensory alterations in the grafted area were assessed both subjectively (patient‐reported difference compared with the contralateral side) and objectively using needle prick and thermal (hot/cold) testing. The pain intensity was measured using the VAS, ranging from 0 (no pain) to 10 (worst possible pain). Patient satisfaction was evaluated using VAS‐based domains, including perceived treatment duration, intraoperative comfort, preoperative anxiety, postoperative swelling, and overall morbidity at 3 months.

### 2.6. Statistical Analysis

Statistical analysis was performed using SPSS version 29 (IBM Corp., Armonk, NY). Sample size calculation was performed a priori using Fisher’s exact test for comparing proportions; assuming survival rates of 95% for autogenous grafts and 75% for bovine grafts, with α = 0.05 and power = 0.80, the required sample size was calculated as 15 patients per group, and 16 patients were enrolled per group to account for potential dropouts. The normality of continuous variables was assessed using the Shapiro–Wilk test, and the homogeneity of variance was verified using Levene’s test. Data are presented as mean ± standard deviation (SD) for normally distributed variables and as median (interquartile range) for non‐normally distributed variables. For variables demonstrating normal distribution (*p*  > 0.05), one‐way analysis of variance (ANOVA) was used to compare means across the three groups. To account for potential confounding factors due to the non‐randomized design, an analysis of covariance (ANCOVA) was performed including the following covariates: anatomical location (maxilla vs. mandible), gingival biotype, age, sex, and baseline ridge width. For variables not meeting normality assumptions, the Kruskal–Wallis test was used as a nonparametric alternative. Graft survival was compared across groups using Fisher’s exact test as the outcome is binary (success/failure) with a fixed 6‐month follow‐up, and 95% confidence intervals for survival proportions were calculated using the Wilson score interval method. The chi‐squared test was used to assess associations between graft type and postsurgical complications, while the Wilcoxon signed‐rank test was used for paired comparisons within groups (e.g., preoperative vs. postoperative patient satisfaction) and the Kruskal–Wallis test was used for between‐group comparisons of patient satisfaction.

## 3. Results

### 3.1. Demographics Information

A total of 48 patients were enrolled and equally allocated into three groups (*n* = 16 per group). Baseline demographic and recipient‐site characteristics are presented in Table 2. Regarding sex distribution, Group I included 12 males and 4 females, Group II included 9 males and 7 females, and Group III included 10 males and 6 females. The anatomical distribution of recipient sites was comparable among the three groups. In Group I, the graft recipient sites included the posterior mandible (*n* = 6, 37.5%), anterior maxilla (*n* = 6, 37.5%), posterior maxilla (*n* = 2, 12.5%), and anterior mandible (*n* = 2, 12.5%). In Group II, recipient sites included the posterior mandible (*n* = 6, 37.5%), anterior maxilla (*n* = 5, 31.3%), posterior maxilla (*n* = 3, 18.8%), and anterior mandible (*n* = 2, 12.5%). Similarly, in Group III, recipient sites included the posterior mandible (*n* = 6, 37.5%), anterior maxilla (*n* = 5, 31.3%), posterior maxilla (*n* = 3, 18.8%), and anterior mandible (*n* = 2, 12.5%). Comparison of recipient‐site distribution among the three groups using the Chi‐square test revealed no statistically significant difference (*p*  > 0.05), indicating that the groups were comparable with respect to the anatomical location of the ridge defects prior to treatment.

**Table 2:** Demographic and clinical characteristics by study group.

Variable	Group I (*n* = 16)	Group II (*n* = 16)	Group III (*n* = 16)	*p*‐Value ^∗^
Sex				
Male *n* (%)	12 (75.0)	9 (56.3)	10 (62.5)	0.53
Female *n* (%)	4 (25.0)	7 (43.7)	6 (37.5)	—
Recipient Site				
Anterior Maxilla *n* (%)	6 (37.5)	5 (31.3)	5 (31.3)	—
Posterior Maxilla *n* (%)	2 (12.5)	3 (18.8)	3 (18.8)	—
Anterior Mandible *n* (%)	2 (12.5)	2 (12.5)	2 (12.5)	—
Posterior Mandible *n* (%)	6 (37.5)	6 (37.5)	6 (37.5)	0.97
Maxilla vs. Mandible				
Maxilla *n* (%)	8 (50.0)	8 (50.0)	8 (50.0)	1.00
Mandible *n* (%)	8 (50.0)	8 (50.0)	8 (50.0)	—
Anterior vs. posterior				
Anterior, *n* (%)	8 (50.0)	7 (43.8)	7 (43.8)	0.90
Posterior, *n* (%)	8 (50.0)	9 (56.2)	9 (56.2)	—

^∗^Fisher’s exact test was used due to small, expected cell frequencies.

Shapiro–Wilk tests confirmed normal distribution for bone formation (*p* = 0.23), bone resorption (*p* = 0.18), and VAS pain scores (*p* = 0.15‐0.41). ANCOVA analyses, adjusting for anatomical location, age, sex, gingival biotype, and baseline ridge width, confirmed the main findings. The adjusted mean differences for bone formation (*p* = 0.580) and bone resorption (*p* = 0.150) remained non‐significant.

### 3.2. Postsurgical Complications

**Figure 5:**
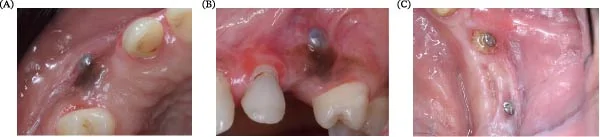
(A, B) Fixation screw shadowing due to thin gingival biotype without bone graft exposure. (C) Screw exposure combined with wound dehiscence and graft necrosis.

The frequency of postsurgical complications is detailed in Table 3. No major postoperative complications were observed during the first 2 weeks. Minor postoperative complications were reported between weeks 3 and 6, specifically nine cases of non‐progressing wound dehiscence. Discomfort during soft tissue examination and palpation was observed in two participants in Group II and 3 in Group III. One case of screwhead exposure occurred in Group I due to a thin gingival biotype; however, no graft exposure was observed (Figure 5). Three cases in Group II and 3 cases in Group III exhibited screw head exposure. Symptomatic screw exposures, which caused soft‐tissue irritation, were removed under topical anesthesia to minimize disruption to the grafts. Following screw removal, spontaneous soft tissue closure occurred without the need for additional flap surgery.

**Table 3:** Postsurgical complications by study group.

Complication	Group I	Group II	Group III	*p*‐Value ^∗^
Wound dehiscence, *n* (%)	1 (6.3)	4 (25.0)	4 (25.0)	0.026
Screw exposure, *n* (%)	1 (6.3)	3 (18.8)	3 (18.8)	0.071
Partial bone loss, *n* (%)	1 (6.3)	4 (25.0)	4 (25.0)	0.026
Sensation alteration (donor site), *n* (%)	2 (12.5)	2 (12.5)	3 (18.8)	0.840
Sensory alteration (donor site), *n* (%)	3 (18.8)	N/A	N/A	—
Graft failure *n* (%)	0 (0)	4 (25.0)	5 (31.3)	0.07^∗∗^

^∗^Fisher’s exact test was used due to small, expected cell frequencies.

^∗∗^Fisher’s exact test.

#### 3.2.1. Bone Graft Success and Integration

Initial wound healing was uneventful in all cases within the autogenous graft group (Group I), with no reported infections or clinical signs of inflammation (survival rate = 100%). In Group II, 12 grafts successfully integrated, resulting in a cumulative survival rate of 75.0% (95% CI: 70.3%–79.7%) (Figure 2). In Group III, 11 grafts successfully integrated, yielding a cumulative survival rate of 69.0% (95% CI: 64.9%–73.1%), *p* = 0.07. The affected grafts were removed under local anesthesia with excision of the granulation tissue. The recipient site was debrided to induce bleeding. Subsequently, the area was closed with soft tissue and managed with antibiotic therapy. Graft failure was observed more frequently in the mandible than in the maxilla; seven failures occurred in the mandible (three in the anterior mandible and four in the posterior mandible) compared to two in the maxilla (one in the anterior maxilla and one in the posterior maxilla). The survival rate for grafts in the maxilla was 91.7% (95% CI: 86.1%–97.3%), compared to 71.0% (95% CI: 63.7%–78.3%) in the mandible, *p* = 0.06). Bone resorption and formation varied across the three study groups (Table 4).

**Table 4:** Bone augmentation measurements (mean ± SD in mm).

Bone augmentation parameter	Group I (*n* = 16)	Group II (*n* = 16)	Group III (*n* = 16)	F‐statistic	*p*‐Value
Native bone thickness	4.50 ± 0.45	4.34 ± 0.39	4.38 ± 0.43	0.61	0.549
Graft thickness	4.50 ± 0.44	4.66 ± 0.40	4.63 ± 0.42	0.61	0.548
Bone formation	3.19 ± 0.44	3.11 ± 0.45	3.06 ± 0.41	0.49	0.614
Bone resorption	1.24 ± 0.43	1.51 ± 0.46	1.50 ± 0.31	2.19	0.122

*Note:* One‐way ANOVA. No statistically significant differences were observed among groups.

#### 3.2.2. Pain and Patient Satisfaction

Postoperative pain scores, assessed using the VAS, are presented in Table 5. All groups reported mild to moderate pain during the first 3 days postoperatively, with peak pain occurring on day 6 in Group I (5.75 ± 1.12) and on day 3 in Groups II (3.06 ± 0.92) and III (3.44 ± 0.81). One‐way ANOVA revealed no significant differences in pain levels among the three groups on day 1 (*p* = 0.843) and day 2 (*p* = 0.061). However, from day 3 through day 7, Group I (autogenous bone graft) experienced significantly higher pain scores compared to Groups II and III (*p*  < 0.001 for all time points). By day 10, pain levels had decreased substantially in all groups, with Group III reporting the lowest scores (0.31 ± 0.48), followed by Group II (0.63 ± 0.50) and Group I (1.00 ± 0.63), with a statistically significant difference among groups (*p* = 0.003). These findings indicate that while all surgical procedures were associated with tolerable postoperative pain, the autogenous bone graft group experienced prolonged and more intense pain, likely attributable to donor site morbidity.

**Table 5:** Visual analog scale pain scale among all groups between day 1 and day 10 after bone graft surgery.

Time point	Group I (*n* = 16)	Group II (*n* = 16)	Group III (*n* = 16)	F‐statistic	*p*‐Value
Day 1	3.25 ± 1.06	3.13 ± 0.81	3.31 ± 0.87	0.17	0.843
Day 2	4.13 ± 1.08	3.38 ± 1.02	3.37 ± 0.88	2.95	0.061
Day 3	4.50 ± 1.03	3.06 ± 0.92	3.44 ± 0.81	9.32	<0.001
Day 4	4.44 ± 1.09	3.13 ± 0.80	3.44 ± 0.72	8.68	<0.001
Day 5	5.31 ± 1.19	2.81 ± 0.75	3.44 ± 0.81	27.88	<0.001
Day 6	5.75 ± 1.12	2.50 ± 0.73	3.00 ± 0.81	53.83	<0.001
Day 7	3.44 ± 0.96	2.06 ± 0.68	2.44 ± 0.72	10.72	<0.001
Day 8	2.19 ± 0.65	1.69 ± 0.71	1.56 ± 0.81	3.27	0.046
Day 9	1.75 ± 0.57	1.31 ± 0.47	1.00 ± 0.63	7.10	0.002
Day 10	1.00 ± 0.63	0.63 ± 0.50	0.31 ± 0.48	6.42	0.003

*Note:* One‐way ANOVA. Shapiro–Wilk tests confirmed normal distribution for VAS pain scores (*p* = 0.15 − 0.41).

Regarding patient satisfaction, Group I reported the lowest satisfaction at 82%, while Groups II and III showed higher satisfaction rates of 89% and 91%, respectively. Notably, all patients in Group III expressed their willingness to undergo the same procedure again if required and indicated that they would recommend it to others.

## 4. Discussion

In this study, participants were allocated to the study groups based on their preference as the authors considered it appropriate to allow patients to select their preferred type of bone graft and to avoid assigning a treatment they might not favor. However, this allocation method does not constitute randomization; accordingly, the study should be classified as a prospective non‐randomized comparative study. The autogenous bone graft group was considered the gold standard, whereas the bovine bone block groups were considered the experimental groups. Autogenous bone block grafting can achieve satisfactory biological outcomes; however, this technique is associated with several drawbacks, including prolonged surgical time, limited graft availability, and risks of damage to adjacent teeth, neurosensory disturbances, donor‐site flap exposure, bleeding, and infection.

The chin was selected as the donor site because it provides an adequate volume of corticocancellous bone with favorable handling characteristics for the reconstruction of the defects included in this study. In addition, the surgical team has experience with symphyseal bone harvesting, allowing predictable graft procurement and adaptation. Although the mandibular ramus is a well‐established alternative donor site and is often preferred to minimize esthetic concerns and reduce the risk of neurosensory disturbances associated with the mental nerve, it may provide a more limited bone volume than that required for the defects treated in the present study. Furthermore, ramus harvesting can present technical challenges depending on the defect morphology and anatomical constraints. Therefore, donor‐site selection was based on the graft dimensions required, the characteristics of the defects under investigation, and the surgical protocol adopted in the current research.

In the present study, these complications were reported, with one biological complication associated with minor wound exposure, which was successfully managed with nonsurgical wound care. Additionally, three cases in the autogenous group developed minor soft tissue sensory alterations at the donor site, secondary to the injury of the peripheral nerve endings of the mental nerve, resulting in an incidence of 18%. Nonetheless, the procedure was associated with higher pain levels and lower patient acceptance compared to alternative methods, primarily due to donor site morbidity and the extended time required for graft harvesting from the chin.

Wound dehiscence at the recipient site was observed more frequently in the noncustomized bovine bone block group compared to the autogenous group, with incidences of 25% and 6%, respectively. Minor bone graft exposure occurred in 25% of cases involving bovine bone grafts, regardless of the type (customized and noncustomized). One study reported higher rates of wound dehiscence and graft exposure (70% and 40%, respectively) [24]. Another study reported wound dehiscence in 33.3% of cases; however, the anatomical location of the grafts was not specified [25]. Bone exposure usually occurs during the early healing phase, primarily due to flap tension over bulky bone grafts. Another contributing factor is the limited blood supply to the distal portion of non‐autogenous bone grafts. This phenomenon was exemplified in the current study by a case where wound dehiscence occurred in a bovine block for 3 months following the surgery. Such complications were more frequently observed in the mandible, likely due to an inadequate vascular supply, which may have contributed to graft migration and impaired site vascularization. These complications have also been documented in other studies [26, 27].

Screwhead exposure was not observed in cases of wound dehiscence, which may be attributed to factors such as graft resorption, a thin gingival phenotype, or a combination of these factors. In some instances, screws were removed, particularly when associated with gingival irritation, especially in cases involving two screws. Screw removal was performed without the need for injectable local anesthesia and did not cause patient discomfort. In the present study, screw exposure occurred in 15% of cases, a significantly lower incidence compared to the 33% reported by others [27]. However, this finding contrasts with results from Angermaier et al. [28], who observed severe complications using equine‐derived cancellous bone blocks, with a failure rate of 60%.

In the current study, the overall failure rate was 18% (9 out of 48 cases), despite the differences in graft material. Notably, failures were more frequently observed in the mandibular recipient sites. This finding may be attributed to the unique anatomical and biological characteristics of the mandible. Compared with the maxilla, the mandible is characterized by a thicker cortical bone layer and reduced vascularity, which may compromise early graft revascularization and integration. Adequate blood supply is essential for graft survival, and the dense cortical architecture of the mandible may delay the remodeling process and increase the risk of graft resorption or incomplete incorporation.

Furthermore, mandibular graft sites, particularly in the posterior region, are subjected to higher occlusal and masticatory forces, which may negatively affect graft stability during the healing phase. Achieving tension‐free soft tissue closure can also be more challenging in mandibular defects because of limited tissue flexibility and shallow vestibular depth in some cases. These factors may predispose the grafted area to wound dehiscence, graft exposure, and subsequent failure.

Another important consideration is the proximity of mandibular surgical sites to vital anatomical structures, such as the inferior alveolar and mental nerves. Postoperative edema, soft‐tissue tension, and surgical manipulation in these regions may contribute to local complications that can indirectly affect the healing environment. Collectively, these anatomical and biomechanical factors may explain the greater susceptibility of mandibular sites to graft failure observed in the present study.

An additional clinical observation was that some patients experienced an altered sensation in the soft tissue covering the grafted area. A total of seven cases were reported, including two cases in Group I located in the posterior mandible, two cases in Group II involving the anterior and posterior maxilla, and three cases in Group III, including one case in the anterior maxilla and two cases in the posterior mandible. Notably, four of the seven cases occurred in posterior mandibular sites.

The altered sensation may be attributed to surgical disruption of periosteal sensory nerve endings during flap elevation and recipient site preparation. The recovery of normal sensation depends on the regeneration of these nerve fibers and the re‐establishment of local neurovascular networks within the healing tissues. In non‐autogenous grafts, particularly bovine‐derived grafts, the absence of viable cellular and neurovascular components may result in a slower regenerative process compared with autogenous bone grafts. Furthermore, the prolonged persistence of graft particles and delayed remodeling characteristics of xenogeneic materials may influence the pattern of soft tissue adaptation and nerve regeneration.

The higher frequency of sensory alterations observed in posterior mandibular sites may also be related to the anatomical proximity of the surgical field to branches of the inferior alveolar and mental nerves as well as the limited soft tissue mobility in this region. Postoperative edema, scar formation, and local tissue tension may further contribute to transient neurosensory disturbances. Although these sensory changes were mild and did not result in significant functional impairment, they represent an important clinical finding that should be considered during treatment planning and patient counseling.

In all cases, the initial mean ridge width increased from 4.35 to 9 mm, with a mean increase of 3.11 ± 0.45 mm after 6 months. These findings align with those reported by Schwarz et al. [24]. However, the present study included a substantially larger sample size for the bovine graft group (32 vs. 9 participants). No significant bone resorption was observed in the customized bovine bone blocks compared to the non‐customized group, suggesting that graft customization has a minimal effect on bone formation and resorption. The resorption rates reported in the present study were significantly lower than previous reports.

A retrospective study examining volume changes in onlay autogenous grafts after implant placement reported mean volume resorption rates of 35% to 51% [29]. In terms of horizontal ridge augmentation, previous studies have documented gains ranging from 4 to 5 mm for autogenous bone blocks and 3.3–5.6 mm for xenogeneic bone blocks [26, 30]. However, these studies did not clearly specify the graft thickness or the associated resorption rates. Resorption of autogenous grafts has been reported to vary widely, ranging from 5.5% to 22% and reaching up to 60% in some cases [26, 31]. Conversely, the clinical performance of freeze‐dried allogenic bone blocks has demonstrated greater variability in outcomes [32–34]. Some investigations have shown significant resorption in cortico‐cancellous fresh frozen block bone allografts after 6 months of healing [35]. Nevertheless, Lumetti et al. [36] observed that fresh‐frozen allogeneic bone block grafts exhibited higher resorption rates compared to autogenous bone (46% vs. 28%), although denser allogeneic bone blocks may achieve more favorable resorption outcomes. Additionally, Aslan et al. [37] reported that the mean increase in horizontal bone was 1.65 mm and the mean resorption rate was 5.39%.

The present study also evaluated postoperative pain using the VAS, revealing clinically relevant differences among the three groups. Patients who received autogenous bone grafts (Group I) reported significantly higher pain scores from day 3 to day 7 compared to both bovine bone block groups (*p*  < 0.001), with peak pain occurring on day 6 (5.75 ± 1.12). In contrast, patients in the noncustomized and customized bovine bone block groups reported consistently lower pain scores throughout the postoperative period, with peak pain occurring earlier (day 3) and at lower intensities (3.06 ± 0.92 and 3.44 ± 0.81, respectively).

This difference is likely attributable to the additional surgical site required for graft harvesting in the autogenous group, which involves a second surgical procedure at the donor site (chin), resulting in increased soft tissue trauma, prolonged healing time, and greater postoperative discomfort [29, 30]. The higher pain levels in the autogenous group may also explain the lower patient satisfaction (82%) compared to the bovine groups (89% and 91%) as pain is a significant determinant of overall patient experience and treatment acceptance. These findings are consistent with previous studies reporting increased morbidity associated with intraoral bone harvesting procedures [31, 32]. Notably, by day 10, pain scores had decreased substantially in all groups, indicating that the acute postoperative pain period is relatively short and manageable with standard analgesic protocols. The lower pain scores observed in the customized bovine bone block group (Group III) compared to the noncustomized group (Group II) may reflect the reduced surgical time and minimized soft tissue manipulation associated with the precise fit of customized grafts, although this difference was not statistically significant at most time points. These findings suggest that xenogeneic bone blocks, particularly customized grafts, may offer a patient‐friendly alternative to autogenous bone harvesting with reduced postoperative pain and faster recovery, which could influence treatment selection in clinical practice.

In the present study, the xenogeneic bone block was combined with contour augmentation using deproteinated bovine bone mineral granules (DBBM) and a concentrated growth factor (CGF) membrane. The use of these materials has been shown to result in clinically significant horizontal bone formation [37, 38]. Therefore, determining the exact contribution of the CXBB to the observed increase in ridge width remains challenging. However, a previous clinical study by Cordaro et al. [26] provided evidence suggesting that augmentation using autogenous bone block grafts in combination with DBBM and a native bilayer collagen membrane reduced bone block resorption. However, they reported a higher incidence of complications in the test groups [26]. Similar findings were noted when autogenous bone blocks were covered solely with DBBM [39].

Recent studies have highlighted the remodeling potential of collagen‐containing xenogeneic bone in horizontal ridge augmentation procedures; however, certain drawbacks have also been observed. Among the complications associated with xenogeneic bone block surgeries, block dehiscence was the most frequently reported and may affect soft tissue sensation due to peripheral nerve exposure [40–42]. Patient satisfaction was the lowest in the non‐autogenous bone graft group. This finding may be interpreted in terms of pain as the autogenous group experienced higher pain levels.

While the present study addressed a relevant and important clinical question, several limitations must be acknowledged, including the short follow‐up period, the non‐randomized design, and the absence of histological evidence. Treatment allocation based on patient preference may have introduced selection bias and potential confounding factors affecting group comparability. Therefore, the findings should be interpreted with caution. A 6‐month follow‐up allows evaluation of early graft healing, integration, and detection of postoperative complications; however, it is insufficient to fully assess long‐term bone remodeling and resorption, which are continuous processes. Accordingly, no conclusions can be drawn regarding long‐term graft stability or implant‐related outcomes based on the present data as implant placement and survival were not evaluated in this study. Future studies with longer follow‐up periods, larger cohorts, and more robust randomized designs are required to confirm these findings and further evaluate the long‐term performance of customized bovine bone grafts in alveolar ridge augmentation.

The clinical implications of this study extend beyond the direct comparison of graft survival rates. The decision to use autogenous versus xenogeneic bone grafts should be individualized based on multiple factors. While autogenous grafts demonstrated superior survival, they were associated with significantly higher postoperative pain (*p*  < 0.001 from day 3 to day 7) and lower patient satisfaction (82%). These findings suggest that the ‘gold standard’ status of autogenous grafts should be reevaluated considering patient‐centered outcomes, particularly when considering that bovine grafts achieved clinically acceptable horizontal augmentation (mean increase: 3.11 ± 0.45 mm) with lower patient morbidity.

The higher failure rates observed in mandibular sites (71% survival) compared to maxillary sites (91.7% survival) have important clinical implications. This finding suggests that more stringent case selection criteria may be warranted for mandibular augmentation procedures, particularly in the posterior region, where occlusal forces are greater. Clinicians should consider the anatomical and biological limitations of the mandible when planning augmentation procedures and may consider additional measures such as extended healing periods, more rigid fixation, or staged approaches in these areas. Customized bovine blocks demonstrated comparable clinical outcomes to non‐customized blocks but were associated with higher patient satisfaction (91%), likely due to reduced surgical time and perceived precision. This finding supports the use of CAD/CAM technology in ridge augmentation procedures, particularly in cases where patient expectations and comfort are prioritized.

## 5. Conclusion

Within the limitations of this prospective comparative study, onlay grafting with bovine bone blocks for horizontal alveolar ridge augmentation demonstrated clinically acceptable outcomes and comparable resorption rates to autogenous bone grafts. However, the observed higher graft survival in the autogenous group (100% vs. 75% vs. 69%) did not reach statistical significance (*p* = 0.07). The wide confidence intervals and relatively small sample size indicate that clinically meaningful differences cannot be ruled out, and larger studies are needed to confirm these findings. Customized bovine bone blocks showed comparable clinical performance to non‐customized blocks in terms of resorption and postoperative complications, with higher patient satisfaction (91%). Given the study’s limitations, including the non‐randomized design, small sample size, and short follow‐up period, these findings should be interpreted cautiously. Larger randomized controlled trials with longer follow‐up are warranted to confirm the comparative efficacy of xenogeneic bone blocks before definitive clinical recommendations can be made.

## Funding

The authors disclosed receipts of financial support for the research graduate studies department at the University of Sharjah.

## Conflicts of Interest

The authors declare no conflicts of interest.

## Data Availability

The data that support the findings of this study are available on request from the corresponding author.
